# Molecular detection and genetic characterization of *Mycoplasma gallisepticum*, *Mycoplama synoviae,* and infectious bronchitis virus in poultry in Myanmar

**DOI:** 10.1186/s12917-019-2018-2

**Published:** 2019-07-25

**Authors:** Sotaro Fujisawa, Shiro Murata, Masaki Takehara, Ken Katakura, Myint Myint Hmoon, Shwe Yee Win, Kazuhiko Ohashi

**Affiliations:** 1 Faculty of Veterinary Medicine, Hokkaido University, Sapporo, Japan; 2 University of Veterinary Science, Yezin, Nay Pyi Taw, Myanmar

**Keywords:** Avian mycoplasmosis, *Mycoplasma gallisepticum*, *Mycoplasma synoviae*, Infectious bronchitis virus, Myanmar

## Abstract

**Background:**

In Southeast Asian countries, including Myanmar, poultry farming is a major industry. In order to manage and maintain stable productivity, it is important to establish policies for biosecurity. Infectious respiratory diseases are a major threat to poultry farming. Avian influenza and Newcastle disease have been reported in Myanmar, but no scientific information is available for other respiratory pathogens, such as mycoplasmas and infectious bronchitis virus (IBV). Identifying the genotypes and serotypes of IBVs is especially important to inform vaccination programs. In this study, we detected *Mycoplasma gallisepticum* (MG), *M. synoviae* (MS), and IBV in several poultry farms in Myanmar.

**Results:**

Samples were collected from 20 farms in three major poultry farming areas in Myanmar, and MG, MS, and IBV were detected on two, four, and eight farms, respectively, by polymerase chain reaction. Phylogenetic analysis revealed that the observed MG and MS isolates were not identical to vaccine strains. Three different genotypes of IBV were detected, but none was an unknown variant.

**Conclusions:**

Mycoplasmas and IBV were detected on poultry farms in Myanmar. Periodic surveillance is required to establish the distribution of each pathogen, and to institute better vaccine protocols.

## Background

Myanmar lies in the western region of mainland Southeast Asia. Agriculture is the backbone of the Myanmar economy, and poultry farming is one of the country’s major industries. In association with the recent economic development of Myanmar, the total number of raised chickens has increased over the last decade [1]. In order to provide a stable supply of poultry products, the development of farm biosecurity measures is required, and it is important that farmers and veterinarians are aware of these measures. Infectious respiratory diseases have severe impacts on the poultry industry. Avian influenza and Newcastle disease are major threats to the poultry industry, and these diseases have been reported in Myanmar [2–4]. Other respiratory pathogens, such as mycoplasmas and infectious bronchitis virus (IBV), have not been investigated in Myanmar, although clinical signs suggesting contagious respiratory diseases have been detected, according to local veterinarians’ observations. These diseases cause considerable economic losses worldwide, and vaccines for their prevention have been developed. It is important to determine the genotypes and/or serotypes of each pathogen circulating in Myanmar to inform vaccination programs.

Avian mycoplasmosis is caused by several pathogenic mycoplasmas. Among them, *Mycoplasma gallisepticum* (MG) and *M. synoviae* (MS) are the most impactful to the poultry industry. MG infections usually cause chronic respiratory disorders and are characterized by sneezing, coughing, and snicks as well as nasal and ocular discharges [5, 6]. MS infections most frequently occur as subclinical upper respiratory tract infections and may cause air sac disease. MS results in infectious synovitis, an acute to chronic infectious disease of chickens [5]. The co-infection by MG or MS with respiratory virus infections, such as IBV and Newcastle disease, can exacerbate the disease conditions [5]. Both MG and MS infections cause considerable economic losses in the poultry industry by reducing weight gains and meat quality in broilers, causing severe drops in egg production in layers, and increasing embryo mortality in breeders [7].

Infectious bronchitis (IB) is a severe acute disease of poultry caused by IBV, which primarily infects the respiratory tracts, with respiratory disease being the most frequent sign. In addition, IBV can infect the kidneys and reproductive tracts and consequently cause kidney damage and decrease in egg production [8]. Generally, IB is controlled by serotype-specific vaccines [9]. The identification of field isolates is necessary for appropriate vaccinations because these vaccines exhibit little cross-reactivity among different serotypes [10, 11].

In this study, we performed molecular detection of MG, MS, and IBV in chickens from poultry farms at the outskirts of three large cities in Myanmar: Mandalay and Pyin Oo Lwin in February 2018 and Yangon in May 2018. In addition, by analyzing genetic characteristics, we detected at least three genotypes of IBV existing in Myanmar. To our knowledge, this is the first report using molecular analysis to detect MG, MS, and IBV in Myanmar.

## Results

### Detection of MG, MS, and IBV in poultry farms in Myanmar

MG, MS and IBV were detected on two, four, and eight farms, respectively (Table 1). Both MG and IBV were detected on farm Ma-4, and both MG and MS were detected on farm Ya-1. On farm Ma-4, where MG and IBV were detected, most sampled chickens showed hypodynamia (Table 1). MS was not detected in Mandalay and Pyin Oo Lwin, while four out of the 10 farms in Yangon were positive for MS. However, IBV was widely detected on the farms in Mandalay and Pyin Oo Lwin, whereas it was not detected in the Yangon area (Table 1). Seasonally, MG and MS were detected in both the wet season (May) and dry season (February), whereas IBV was detected only in the dry season (Table 1).

**Table 1:** Details of the distribution of avian pathogens

Sampling area	Farm ID	Date	No. of chickens	No. of detected/No. of tested^a^ (%)				Note
				MG	MS	IBV	Genotype of IBV	
Mandalay	Ma-1	Feb. 10, 2018	12	0/4 (0.0)	0/4 (0.0)	2/4 (50.0)	JP-1	
	Ma-2	Feb. 10, 2018	9	0/3 (0.0)	0/3 (0.0)	3/3 (100)	Mass	
	Ma-3	Feb. 10, 2018	9	0/3 (0.0)	0/3 (0.0)	2/3 (66.7)	JP-1	
	Ma-4	Feb. 11, 2018	9	3/3 (100)	0/3 (0.0)	3/3 (100)	Mass	Most chicken showed hypodynamia and diarrhea.
	Ma-5	Feb. 11, 2018	9	0/3 (0.0)	0/3 (0.0)	1/3 (33.3)	JP-1	
Pyin Oo Lwin	Py-1	Feb. 12, 2018	9	0/3 (0.0)	0/3 (0.0)	1/3 (33.3)	JP-1	
	Py-2	Feb. 12, 2018	9	0/3 (0.0)	0/3 (0.0)	1/3 (33.3)	ND^b^	
	Py-3	Feb. 12, 2018	9	0/3 (0.0)	0/3 (0.0)	0/3 (0.0)	–	
	Py-4	Feb. 12, 2018	9	0/3 (0.0)	0/3 (0.0)	0/3 (0.0)	–	
	Py-5	Feb. 12, 2018	9	0/3 (0.0)	0/3 (0.0)	1/3 (33.3)	JP-2	A chicken showed torticollis, and another chicken showed deformed leg.
Yangon	Ya-1	May 28, 2018	9	3/3 (100)	2/3 (66.7)	0/3 (0.0)	–	A chicken showed facial swelling.
	Ya-2	May 28, 2018	6	0/2 (0.0)	0/2 (0.0)	0/2 (0.0)	–	
	Ya-3	May 28, 2018	6	0/2 (0.0)	0/2 (0.0)	0/2 (0.0)	–	Some chickens showed diarrhea.
	Ya-4	May 28, 2018	6	0/2 (0.0)	0/2 (0.0)	0/2 (0.0)	–	
	Ya-5	May 29, 2018	9	0/3 (0.0)	1/3 (33.3)	0/3 (0.0)	–	
	Ya-6	May 29, 2018	6	0/2 (0.0)	1/2 (50.0)	0/2 (0.0)	–	
	Ya-7	May 29, 2018	9	0/3 (0.0)	0/3 (0.0)	0/3 (0.0)	–	
	Ya-8	May 29, 2018	9	0/3 (0.0)	1/3 (33.3)	0/3 (0.0)	–	
	Ya-9	May 29, 2018	9	0/3 (0.0)	0/3 (0.0)	0/3 (0.0)	–	
	Ya-10	May 29, 2018	9	0/3 (0.0)	0/3 (0.0)	0/3 (0.0)	–	
Total				6/57 (10.5)	5/57 (8.8)	14/57 (24.6)		

^a^ Three samples of the oropharyngeal swabs were pooled and analyzed

^b^ Sequence was not detected because of the low concentration of the template cDNA

### Phylogenetic analysis of MG, MS, and IBV

**Fig. 1:**
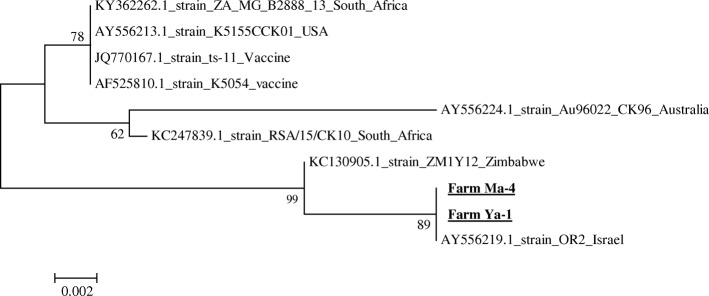
A phylogenetic tree based on the alignment of the nucleotide sequences of the *gapA* gene in isolated and reference strains of *M. gallisepticum*. The tree was built with the neighbor-joining method using the MEGA 6.0 software. Numbers indicate bootstrap percentages (1,000 replicates). The scale indicates the divergence time

**Fig. 2:**
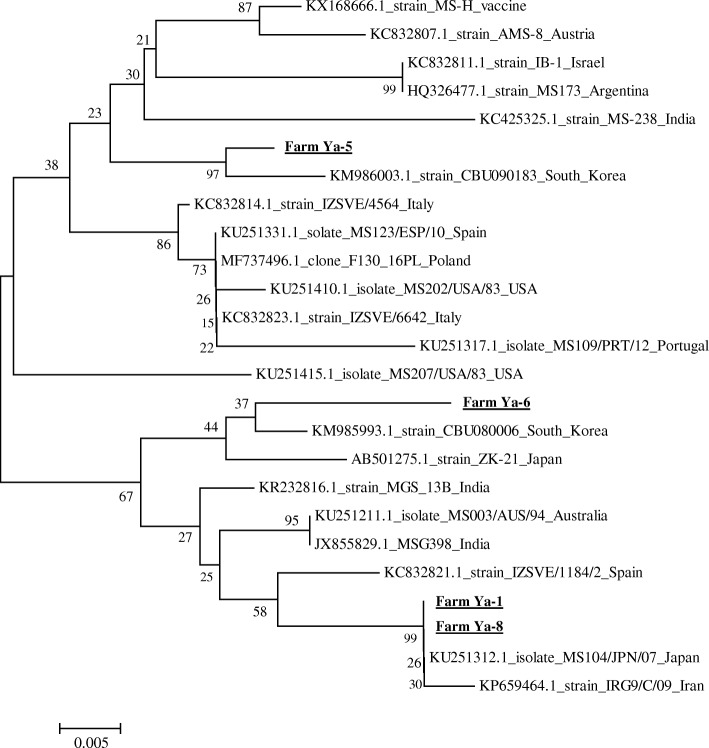
A phylogenetic tree based on the alignment of the nucleotide sequences of the *vlhA* gene in isolated and reference strains of *M. synoviae.* The methodology was the same as that for *M. gallisepticum* in Fig. 1

**Fig. 3:**
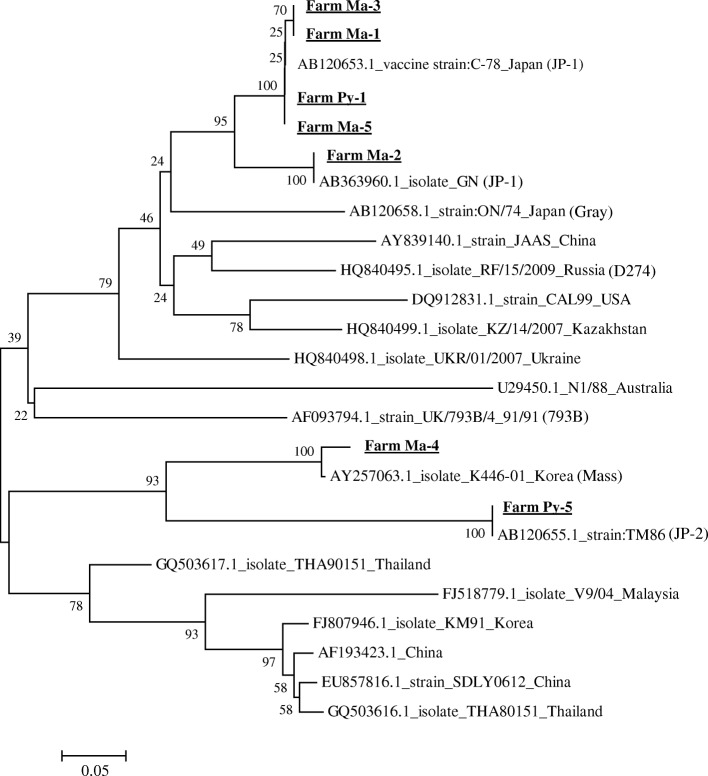
A phylogenetic tree based on the alignment of the nucleotide sequences of the *S1 glycoprotein* gene in isolated and reference strains of infectious bronchitis virus. The methodology was the same as that for *M. gallisepticum* in Fig. 1

The phylogenetic tree for MG revealed that the two MG isolates detected in farm Ma-4 and Ya-1 were genetically identical; however, they were classified in a different cluster than those of vaccine strains (Fig. 1). All the detected MS isolates were not identical to vaccine strains. The detected MS isolates were closely related to strains isolated in East Asian countries (South Korea and Japan) (Fig. 2). No unknown variants were detected in the observed IBV isolates (Fig. 3). The IBV isolates detected in farms Ma-1, Ma-3, Ma-5, and Py-1 were closely related to a vaccine strain, C-78, and IBV isolates detected in farms Ma-2, Ma-4, and Py-5 were similar to IBV strains GN, K446–01 and TM86, respectively (Table 1, Fig. 3).

## Discussion

Respiratory diseases cause significant economic losses in the poultry industry worldwide. Poultry farming is an important industry in Southeast Asian countries, including Myanmar. Nevertheless, surveillance of the distribution of such pathogens causing respiratory diseases is lacking in Myanmar. According to the observations by local veterinarians, respiratory signs that are indicative of IB or mycoplasmosis have been observed in poultry farms in Myanmar. We detected MG, MS, and IBV in poultry farms in Myanmar.

In this study, MG was sporadically detected regardless of the area and season, whereas MS was detected only in the Yangon area in May. In addition, each bacteria strain detected in this study was genetically different from vaccine strains. Furthermore, the *vlhA* genes of all the MS strains were closely related to those strains isolated in East Asian countries, such as South Korea and Japan, indicating that endemic strains from Asian countries may be prevalent in the poultry farms in Myanmar. Appropriate vaccination programs are necessary to prevent diseases caused by mycoplasmas, because both MG and MS can spread easily through vertical and horizontal transmission [6, 12].

We found that IBV was widely distributed in the two districts around the center of Myanmar (Mandalay and Pyin Oo Lwin) in this study. In contrast, IBV was not detected at farms in the Yangon, the southern area of the country. Samples were collected in Yangon in May, which is the wet season in Myanmar, whereas sampling in Mandalay and Pyin Oo Lwin was conducted in February (the dry season). The climates in Myanmar are largely different in each season and in each area. Therefore, surveillance should be periodically performed throughout the country over the course of the year, especially considering the seasons.

Phylogenetic analysis revealed that the *S1* gene of the IBVs detected at the Ma-1, Ma-3, Ma-5, and Py-1 farms were closely related to that of the C-78 IBV vaccine strain and that the IBV detected at farm Ma-2 was close to strain GN (both are classified as JP-1 types [13, 14]). The isolate at farm Ma-4 was close to K446–01 (Mass type [13]), and the isolate from farm Py-5 was close to TM86 (JP-2 type [13]). All the IBV types detected are widely distributed [13, 14], and vaccines for their attenuated strains are commercially available. In this survey, we found that the JP-1, JP-2, and Mass types of IBV were present in poultry farms in Myanmar. According to local veterinarians, vaccines with Mass, D274 and 793B serotypes are mainly used to prevent the incidence of IB disease in Myanmar. It is possible that the IBVs detected at farm Ma-4 were derived from attenuated vaccine strains. The fact that other types of IBVs were detected in Myanmar is important for the identification of appropriate vaccines, because vaccines for different types of IBVs do not cross-protect. Therefore, periodical surveillance using larger samples is required to identify the distributions and types of IBVs circulating in Myanmar.

## Conclusions

This is the first report showing the presence of MG, MS and IBV in poultry farms in Myanmar. Several genotypes of IBV were detected in Myanmar, and some of them appear to be genetically different from the vaccine strains currently used in Myanmar. To improve vaccination programs and reduce the economic losses caused by these devastating pathogens, periodic surveillance including appropriate sample sizes should be performed. In addition, some agents threatening the poultry industry include enteropathogens, oncogenic viruses, and other respiratory pathogens. Further study is necessary to assess the prevalence of infectious agents and develop control strategies..

## Methods

### Sample collection

Chicken oropharyngeal swabs were collected from five farms in Mandalay and five farms in Pyin Oo Lwin in February 2018, and 10 farms in Yangon in May 2018. Swab samples were collected from six, nine or twelve chickens in each farm (Table 1), and all samples were collected from adult laying hens. The sample collection was carried out after we obtained informed consent from farm owners. The breeds of chickens were Rhode Island Red and White Leghorn. Samples were transferred to the laboratory in cool condition as dry swabs within 2 days, and kept at − 80 °C until use. Three swabs taken from each chicken were pooled and used for DNA/RNA extraction, following polymerase chain reaction (PCR) detection and sequence analysis.

### Nucleic acid extraction and cDNA synthesis

DNA was extracted from pooled swab samples using a QIAamp DNA Mini Kit (Qiagen, Hilden, Germany) according to the manufacturer’s instructions, and the samples were stored at − 20 °C until use. For RNA extraction, a FastGene RNA Premium/Basic Kit (NIPPON Genetics Co. Ltd., Tokyo, Japan) was used according to the manufacturer’s instructions. cDNA was synthesized using PrimeScript™ Reverse Transcriptase (TaKaRa Bio Inc., Shiga, Japan) as directed by the manufacturer.

### PCR

The DNA and cDNA samples were used as templates for PCR analysis. MG, MS, and IBV were detected by amplifying the *pMGA1.2* (hemagglutinin protein) gene, the MS2/12 DNA fragment of *vlhA* (variable lipoprotein hemagglutinin A gene), and the *S1* (the spike glycoprotein) gene, respectively. The primer sequences used in each amplification and the PCR conditions are summarized in Table 2. For MG and MS, nested PCRs were performed. The PCR mixture contained 10 pmol of each primer, 1 U of TaKaRa Ex Taq (TaKaRa Bio Inc.), and 200 μM of each deoxynucleotide (TaKaRa Bio Inc.).

**Table 2:** Primers used for amplification of each gene in this study

Target gene	Primer name	Primer sequences (5′ – 3′)	Size in nucleotides (bp)	References
For detection of each pathogen				
pMGA1.2				
1st	pMGAFo	GTG AAG AAA AAA AAC ATA TTA AAG TTT	1,900	Mardassi et al., 2005 [15]
	pMGARo	CTA AGA TGG ATT TGA AAC ATT AGT		
2nd	pMGAF1i	CTA GTT AAT ACT AGT GAT CAA GTG AAA CTA	500	Mardassi et al., 2005 [15]
	pMGAR1i	TTG AAC ATT GTT CTT TGG AAC CAT CAT		
MS2/12				
1st	MS1.2Fo	AAA CTA CAA AAC TTT GTA ATG GCT	1,200	Mardassi et al., 2005 [15]
	MS1.2Ro	TTA CAA GTA CGG TGT TTA ATC AAT		
2nd	MS1.2F1i	ATT ACC AAG CAG ATG GTT ACG ACG T	450	Mardassi et al., 2005 [15]
	MS1.2R2i	AGT TAT AGT AAC TCC GTT TGT TCC A		
*S1*	IBV-S1	AGG AAT GGT AAG TTR CTR GTW AGA G	620–640	Mase et al., 2004 [16]
	IBV-S2	GCG CAG TAC CRT TRA YAA AAT AAG C		
For sequence analysis				
*gapA*	gapA 3F	TTC TAG CGC TTT AGC CCT AAA CCC	332	Ferguson et al., 2005 [17]
	gapA 4R	CTT GTG GAA CAG CAA CGT ATT CGC		
*vlhA*	vlhA f	TAC TAT TAG CAG CTA GTG C	350 / 400	Dijkman et al., 2016 [18]
	vlhA R	AGT AAC CGA TCC GCT TAA T		

### Sequence analysis

To analyze the genetic characteristics of MG and MS, the *gapA* gene and the DNA fragment of *vlhA* gene were amplified using a PCR assay and sequenced. For sequencing, the amplicons were purified using a FastGene gel/PCR extraction kit (NIPPON Genetics Co. Ltd.), and the nucleotide sequences were determined using the GenomeLab™ GeXP Genetic Analysis System (Beckman Coulter, Fullerton, CA, USA). The resulting sequences of the *gapA*, *vlhA*, and *S1* genes were aligned using MEGA6 software [19], and the phylogenetic trees were constructed with the same software using the neighbor-joining method [20].

## Data Availability

The datasets supporting the conclusions of this article are available from the corresponding author on reasonable request.

## Abbreviations

IB: Infectious bronchitis
IBV: Infectious bronchitis virus
MG: *Mycoplasma gallisepticum*
MS: *Mycoplasma synoviae*
PCR: Polymerase chain reaction
